# Vitamin B6 inhibits activity of *Helicobacter pylori* adenylosuccinate synthetase and growth of reference and clinical, antibiotic-resistant *H. pylori* strains

**DOI:** 10.1080/14756366.2024.2372734

**Published:** 2024-08-16

**Authors:** Marta Ilona Wojtyś, Weronika Maksymiuk, Marta Narczyk, Ante Bubić, Ivana Leščić Ašler, Paweł Krzyżek, Grażyna Gościniak, Elżbieta Katarzyna Jagusztyn-Krynicka, Agnieszka Bzowska

**Affiliations:** a Division of Biophysics, Institute of Experimental Physics, Faculty of Physics, University of Warsaw, Warsaw, Poland; b Department of Bacterial Genetics, Institute of Microbiology, Faculty of Biology, University of Warsaw, Warsaw, Poland; c Division of Physical Chemistry, Ruđer Bošković Institute, Zagreb, Croatia; d Department of Microbiology, Faculty of Medicine, Wroclaw Medical University, Wroclaw, Poland

**Keywords:** Adenylosuccinate synthetase, *Helicobacter pylori*, vitamin B6, antimicrobial activity, X-ray structure

## Abstract

The current therapies against gastric pathogen *Helicobacter pylori* are ineffective in over 20% of patients. Enzymes belonging to the purine salvage pathway are considered as novel drug targets in this pathogen. Therefore, the main aim of the current study was to determine the antibacterial activity of pyridoxal 5’-phosphate (PLP), an active form of vitamin B6, against reference and clinical strains of *H. pylori*. Using a broad set of microbiological, physicochemical (UV absorption, LC-MS, X-ray analysis) and *in silico* experiments, we were able to prove that PLP inhibits adenylosuccinate synthetase (AdSS) from *H. pylori* by the competition with GTP (IC_50_^eq^ ∼30 nM). This behaviour was attributed to formation of a Schiff base with a lysine residue (a covalent bond with Lys322 in the GTP binding site of AdSS) and was potentiated by the presence of vitamin C. This antibacterial activity of PLP gives hope for its future use against *H. pylori*.

## Introduction

*Helicobacter pylori*, a Gram-negative microaerophilic bacterium, was first discovered in 1984 by Marshall and Warren^1^. Since then, it has gained significant recognition both in the scientific community and among the general public, what was strongly associated with its potential to cause severe health problems. *H. pylori* is known to colonise and persist in the human stomach^2^. The prevalence of infection varies across countries and age groups, although it is estimated that approximately half of the world’s population is affected by this bacterium^3^^,^^4^. Fortunately, most strains of *H. pylori* do not cause clinical symptoms^4^. Even though, certain strains can lead to the development of serious diseases of a digestive system, such as chronic active gastritis, peptic ulceration, gastric adenocarcinoma, and gastric mucosa-associated lymphoid tissue (MALT) lymphoma^5^. As a result, *H. pylori* is considered a significant threat to human health and has been classified as a class I human carcinogen^6^.

The current treatment of *H. pylori* infections involves the administration of multiple drugs that target different molecular pathways^7^^,^^8^. However, these therapies are ineffective in over 20% of patients^4^. Furthermore, there is a growing concern regarding the development of antibiotic resistance in *H. pylori* strains, with some demonstrating simultaneous resistance to multiple drugs, including amoxicillin, metronidazole, and clarithromycin^9^^,^^10^. Given these issues, it is crucial to explore alternative molecular mechanisms for drug development to effectively eradicate *H. pylori*.

Enzymes belonging to the purine salvage pathway are potential targets for novel drugs against *H. pylori*, as recent studies have shown that this bacterium is unable to synthesise purine nucleotides *de novo*. The lack of some essential enzymes in the *de novo* purine synthesis pathway is the reason behind this limitation^11^. Consequently, to obtain essential DNA and RNA building blocks, *H. pylori* has to recover purines and purine nucleotides from its environment. Because of the significance of purine production and its direct effect on bacterial growth rates, targeting enzymes of the purine salvage pathway has emerged as a promising approach for discovering new drugs against *H. pylori*^12–15^.

Adenylosuccinate synthetase (AdSS, EC 6.3.4.4) is an ubiquitous enzyme that catalyses the biosynthesis of adenylosuccinate (AMPS) from inosine-5′-monophosphate (IMP), L-aspartate (Asp) and guanosine-5′-monophosphate (GTP)^15^:

IMP + Asp + GTP(Mg2+)⇔AMPS + GDP + Pi

The research conducted by Liechti and Goldberg^11^ revealed that the *H. pylori* G27 strain, lacking the purine salvage pathway enzyme AdSS (a *purA* gene deletion), could only grow in the presence of adenine or adenosine supplemented medium. Furthermore, its growth was notably hampered in a medium abundant in nutrients. These results strongly suggest that AdSS is critical for the survival of *H. pylori*.

Our previous research endeavours have resulted in the acquisition of the recombinant AdSS from *H. pylori* 26695 strain, with subsequent determination of its three-dimensional structure and in-depth analysis of interactions with an influential inhibitor, hadacidin^13^^,^^14^^,^^16^. However, despite noteworthy inhibitory effects of hadacidin against AdSS of *H. pylori*, it fails to impede the bacterial multiplication of *H. pylori*^17^. Aiming to elucidate the mechanism underlying inability of hadacidin to hinder *H. pylori* proliferation, we employed a comprehensive method to evaluate the cellular uptake of this and other compounds by bacterial cells^17^. Our findings revealed a lack of penetration of hadacidin into *H. pylori* cells^17^. While these results do not wholly eliminate the possibility of utilising AdSS inhibitors to combat *H. pylori*, they do suggest that alternative potent inhibitors of AdSS should be explored and thoroughly assessed for their efficacy in the development of novel drugs against *H. pylori* infection.

**Figure 1.**
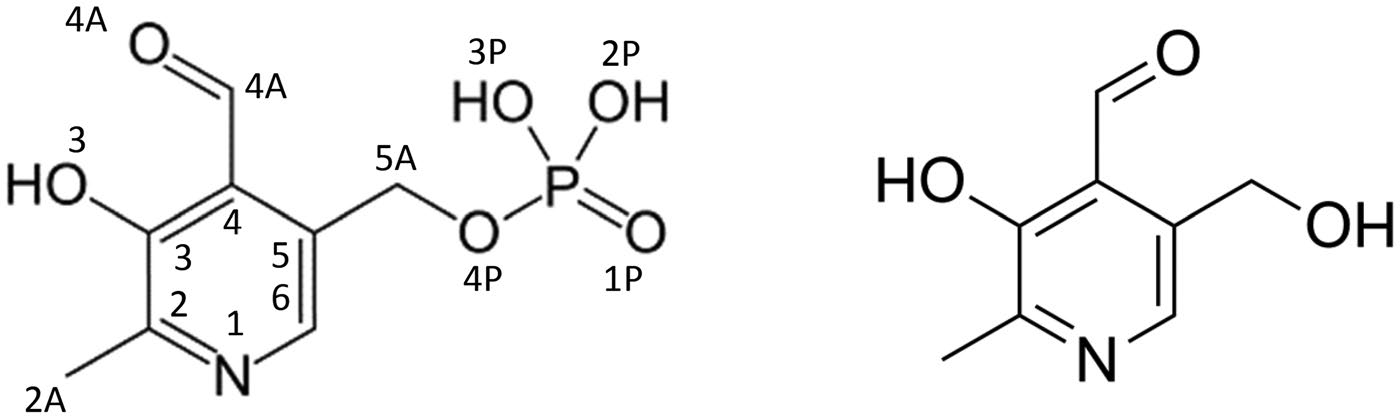
Scheme of pyridoxal 5’-phosphate (PLP, left), with atom numbering as in the PDB file 8QWA, and of one of its metabolic precursors - pyridoxal.

Dong and Fromm^18^ have shown that pyridoxal 5′-phosphate (PLP, Figure 1), which is the active form of the vitamin B6, leads to almost complete inactivation of AdSS from *Escherichia coli* through the formation of a Schiff base with a specific lysine residue. Interestingly, among all AdSS substrates, only GTP in high concentrations is capable of providing complete protection from this inactivation. This suggests that PLP competes with GTP for binding to the active site of AdSS.

In this study, we characterised the inhibition of AdSS from *H. pylori* 26695 strain by PLP. Additionally, we examined the impact of PLP and its metabolic precursors on the replication of several *H. pylori* strains: wild-type 26695, N6, P12, as well as three clinical strains resistant to clarithromycin, metronidazole and both drugs simultaneously. Furthermore, we successfully obtained the three-dimensional structure of the PLP complex with AdSS from *H. pylori* 26695 and characterised the interactions of the inhibitor with the enzyme’s active site.

## Materials and methods

### AdSS protein purification

Adenylosuccinate synthetase (AdSS) from *H. pylori* 26695 and its’ C-His tag variant were obtained as previously described^14^^,^^16^. The gene coding for AdSS (*purA*) was isolated from genomic DNA of *H. pylori* strain ATCC 26695 (obtained from the collection of the Department of Bacterial Genetics, Institute of Microbiology, Faculty of Biology, University of Warsaw, Poland) and amplified using the Phusion High-Fidelity PCR kit (New England BioLabs, Ipswich, MA) with a set of specific DNA primers for both the 5′ and the 3′ end of the gene, and ligated into the NdeI and XhoI restricted *pET21b* expression vector (Invitrogen, Carlsbad, CA). From the resulting *pET21b-HPpurA* plasmid, the plasmid variant coding for AdSS with -LEHHHHHH addition at protein’s C-terminus was prepared by inversed PCR, using primers 5′-TAGAAAAATCGTGTCTTCTC-3′ and 5′-CTCGAGCACCACCAC-3′, and Phusion Flash High-Fidelity PCR Master Mix 2X (New England Biolabs), according to manufacturer’s instructions. Both plasmids, *pET21b-HPpurA* and *pET21b-HPpurA-His*, were (separately) subcloned by electroporation into *Escherichia coli* cell strain BL21-CodonPlus(DE3)-RIL (Agilent Biotechnology, Santa Clara, CA) which was used for protein expression. Protein expression was performed in the LB medium containing 100 mg/mL of ampicillin, after the induction with 0.5 mM IPTG (Isopropyl β-D-1-thiogalactopyranoside) and proceeded overnight at 18 °C and 220 rpm.

For purification of the wild-type AdSS, three chromatographic steps were used^16^. After cell lysis and cell debris separation by centrifugation, the protein extract was subjected to a cation-exchange chromatography. SP-Sepharose FF column was used (55 mL), with a flow rate of 75 mL/h, and equilibrated in 50 mM phosphate buffer pH 6.5 containing 2 mM EDTA and 1 mM DTT. All bound proteins (including AdSS) were eluted with 1 M NaCl in the starting buffer. Concentrated combined fractions containing AdSS were loaded onto a size-exclusion chromatography (SEC) column (190 ml) of Sephacryl S-200 HR, equilibrated in 50 mM phosphate buffer pH 6.5 containing 150 mM NaCl, 2 mM EDTA and 1 mM DTT, and operated at 11 mL/h. AdSS-containing fractions were pulled, concentrated and transferred to 50 mM Tris–HCl buffer pH 8.5 containing 1 mM DTT. The sample thus prepared was loaded onto the anion exchange chromatography column MonoQ 5/50 GL on the ÅKTA protein purification system (Cytiva Life Sciences, Marlborough, MA). The column was equilibrated in 50 mM Tris–HCl buffer pH 8.5 containing 1 mM DTT and elution was performed by a linear gradient of NaCl concentration in the same buffer. During this chromatography step, the flow rate was maintained at 60 mL/h. The final sample of AdSS for use in further experiments was prepared by concentrating the enzyme to ∼15 mg/mL and transferring it to 20 mM Hepes-NaOH buffer pH 6.5 containing 2 mM TCEP (tris(2-carboxyethyl)phosphine), and stored in aliquots at −80 °C.

Addition of His-tag greatly simplified the purification procedure of C-His-AdSS^14^. Three mL of Ni-NTA agarose (Protino, Macherey-Nagel, Düren, Germany) equilibrated in 50 mM Na-phosphate buffer pH 8.0 containing 0.5 M NaCl and 10 mM imidazole (buffer A) was added to the protein extract after cell lysis. After 30 min of incubation, agarose (with bound AdSS) was poured into the purification column and washed with 20 mL of buffer A. After another washing step (20 mL of buffer A, but with 20 mM imidazole), bound proteins were eluted with 9 mL of buffer A with 300 mM imidazole. Additionally, SEC of proteins eluted with 300 mM imidazole was performed on a Sephacryl S-200 16/60 column (Cytiva Life Sciences), equilibrated in 20 mM Hepes buffer pH 7.0 containing 150 mM NaCl and 1 mM β-mercaptoethanol, and operated at 0.6 mL/min on the ÅKTA protein purification system. The final sample of C-His-AdSS for use in further experiments was prepared by concentrating the enzyme to ∼15 mg/mL, and stored in aliquots at −80 °C.

Because both variants have very similar kinetic properties, and for the results of enzyme inhibition tests, it basically does not matter which of them is used^14^, their usage was made interchangeably. In the X-ray structural studies, the C-His tag variant was used as it crystallised better. The C-His tag is located on the flexible protein end, far away from the active site, hence it probably has no influence on the interactions of ligands with the enzyme^14^.

### Materials

AdSS substrates (GTP, IMP, aspartate), chemicals used to measure AdSS activity, namely Hepes and Tris, TCEP, MgSO_4_ and MgCl_2_, PLP, its precursors and ascorbic acid (vitamin C) were from Sigma-Aldrich (Saint Louis, Missouri, USA). Since PLP (Sigma P3657) contains unknown number of crystalline water molecules, prior to use the concentration of PLP solutions was always determined spectrophotometrically using the extinction coefficient of 4900 M^−1 ^cm^−1^ at 388 nm at pH 7^19^. Although the spectra were recorded for aqueous solutions, minor alterations towards the spectrum at pH 7 are anticipated, as PLP is protonated at the O3 position and deprotonated at the PLP-N1 position under these conditions (refer to Figure 1), owing to the respective pKa values of ∼9 and ∼5.8^20^.

The *H. pylori* strains utilised in this study were wild-type strains 26695 and P12^21^, obtained from ATCC (Manassas, Virginia, USA), and strain N6 from Pasteur Institute in Paris, France^22^.

Clinical strains, M92 – resistant to clarithromycin (CLR), M93 – resistant to metronidazole (MTZ), and M26 and M91 – resistant to both drugs, were obtained from primary infected (non-eradicated) patients suffering various stomach-related conditions. These strains were isolated and characterised in the period 2015–2020 as reported by Krzyżek et al.^23^ and are now part of the Department of Microbiology collection at Wroclaw Medical University in Poland.

Reagents and microplates used for bacterial culture experiments, including checking for the cellular uptake of PLP and pyridoxal in the form of hydrochloride (PI-h) by *H. pylori*, were applied as described in Narczyk et al.^13^ and Wojtyś et al.^17^

The reagents used for crystallisation of AdSS complex with PLP, including Tris, HCl, PEG 4000, PEG 3350, Am_2_SO_4_, Li_2_SO_4_ and glycerol, were sourced from Sigma-Aldrich (Saint Louis, Missouri, USA) and Carl Roth GmbH + Co. KG (Karlsruhe, Germany).

### Enzyme activity assay

The AdSS activity was measured at 25 °C as previously described^13^^,^^16^. A reaction mixture contained 20 mM Hepes/NaOH buffer at pH 7.7, or 20 mM Tris/HCl buffer pH 7.7, 1 mM MgSO_4_ and enzyme substrates. To measure the specific activity, saturating substrate concentrations were used, 0.06 mM GTP, 0.15 mM IMP and 5 mM Asp. The enzyme concentration in the reaction mixture was typically 20 nM, expressed in enzyme subunits. The molecular mass of one AdSS subunit is 45.7 kDa for the WT enzyme, and 46.8 kDa for the C-His tag variant (as calculated by the ProtParam tool on the ExPASy server^24^).

The reaction volume was either 1 mL or 1.4 mL, for a path length of 1 cm semi-micro cuvettes (1 cm × 0.4 cm) or 0.5 cm standard cuvettes (0.5 cm × 1 cm), respectively. The choice of cuvette was made to ensure that the initial absorbance at the observation wavelength of 280 nm did not exceed 1.1. Reaction mixture was equilibrated for 5 min at 25 °C before the AdSS was added, and then absorbance changes at 280 nm were followed for 3 min. To check if the reaction proceeds linearly (the absorbance change vs. time indeed represents the initial velocity), the straight line was fitted to the three reaction segments, namely, 0.7–1.2 min, 1.0–1.5 min and 1.2–1.7 min, and for each reaction the average of these three values was used for the following data analysis.

GraphPad Prism 9 program (Graphpad Software, Boston, MA, USA) was used for all calculations regarding enzyme activity and enzyme inhibition.

Extinction coefficient change at 280 nm of 1.17 × 10^4^ M^−1 ^cm^−1^ (corresponding to the formation of adenylosuccinate)^25^ was used to calculate concentration of the product formed. One unit (U) of AdSS specific activity is defined as µmol of adenylosuccinate formed per min at 25 °C. Specific activity is expressed as units per mg of protein (U/mg).

The inhibition of AdSS by PLP and its precursors was investigated to determine IC_50_ and the inhibition constant, K_i_ of the initial enzyme-inhibitor complex, as well as to assess potential competition with the enzyme substrates.

The determination of IC_50_ was carried out at saturating substrate concentrations (0.06 mM GTP, 0.15 mM IMP, 5 mM Asp) and PLP concentration in the range 0–60 μM. The enzyme activity vs. inhibitor concentration (A([I])) was analysed using the four-parameter dose response curve equation which involved consideration of the Hill parameter (h)^26^:

(1) $$A(\left[I\right])=\frac{\left(Ao-A\infty \right)}{1+10h(\text{log}[I]-\text{log}\;IC50)}+A\infty$$

The inhibition model and inhibition constant, K_i_ of the initial PLP-AdSS complex were determined by varying concentrations of GTP (ranging from 5–60 μM) and PLP (0–12 μM) while ensuring saturation of IMP (at 0.15 mM) and Asp (at 5 mM). The data obtained was analysed using the GraphPad Prism 9 software, which allowed various inhibition models to be fitted globally to the data to determine the type of inhibition and inhibition constant. The most suitable model was selected using the extra sum-of-squares F test for models with varying parameters and Akaike’s criterion (AIC) for non-nested models as described by Narczyk et al.^13^

### Time-dependent inactivation of AdSS by PLP

The enzyme (0.022 μM subunits) was incubated for a specific time period at 25 °C within a reaction mixture comprised of 22.2 mM Hepes/NaOH at pH 7.7, 1.11 mM MgSO_4_, 5.55 mM Asp, 167 μM IMP and varying concentrations of PLP within the range of 0.03 μM to 8.1 μM. To initiate the reaction, 100 μL of 0.6 mM GTP was added to 900 μL of aforementioned reaction mixture. Final concentration of substrates, buffer and MgSO_4_ matched those used in the reaction mixture for the specific activity measurements. In this way, the incubated reagents of the reaction mixture were diluted only slightly with the addition of GTP (in a 10:9 ratio) to avoid inactivation of the resulting Schiff base formed between PLP and AdSS.

The reaction initiated by the addition of GTP was followed spectrophotometrically at 280 nm as described in the “*Enzyme activity assay”* section to measure the activity (A) of AdSS dependence over time (t) of incubation for each of the PLP concentrations studied. The one-phase exponential decay was fitted to each of the A(t) traces, as follows:

(2) A(t)=Aoexp (− t k)+Aeq

where A_o_ is the activity at the start of the incubation (with no PLP-AdSS complex yet formed), k is the rate constant of the inactivation process and A_eq_ is a residual activity observed when equilibrium of the AdSS-PLP complex formation is reached. Half-time, t_1/2_ of the inactivation process is calculated as t_1/2_ = ln(2)/k. Activity observed in equilibrium, A_eq_, vs. inhibitor concentration [I] was then analysed to determine IC_50_^eq^ in these conditions by fitting Equation (1).

### Reduction of the Schiff base by vitamin C

In these experiments, vitamin C was present during inactivation of AdSS by PLP or was added after the inactivation process reached the equilibrium. In the first case, 4.15 μM of the enzyme (in terms of subunits) was incubated with 100 μM of PLP and 1 mM of vitamin C in 20 mM Hepes/NaOH buffer at pH 7.7 and 1 mM of MgSO_4_ at 25 °C. From this incubation mixture, aliquots of 5 μL were collected at specific time intervals and added to cuvettes with path-length of 1 cm, containing 1 ml of the reaction mixture (20 mM Hepes/NaOH buffer pH 7.7, 1 mM MgSO_4_, 0.06 mM GTP, 0.15 mM IMP and 5 mM Asp). The reaction was followed spectrophotometrically at 280 nm and enzyme activity was determined from absorbance change as described in the “*Enzyme activity assay”* section.

In the second case, experimental procedure entailed the incubation of 5.0 μM of the enzyme (in terms of subunits) with 100 μM of PLP in 20 mM Hepes/NaOH buffer at pH 7.7, and 1 mM of MgSO_4_ at 25 °C. After 70 min vitamin C was added to the mixture in the final concentration of 0.17 mM or 1.67 mM. This resulted in a dilution of the enzyme to 4.15 μM, following which incubation was continued for an additional 1 h and 50 min. To monitor changes in enzyme activity, aliquots of 4 μL and 5 μL were withdrawn at specific time intervals before and after the addition of vitamin C, respectively. The activity of the enzyme was measured using the same method as in the first approach.

In both approaches, samples treated in the same way but without addition of vitamin C were analysed as a control.

### Crystallisation of AdSS-PLP complex and diffraction studies

C-His tag AdSS, 15 mg/mL in 20 mM Hepes/NaOH buffer at pH 7.0, 150 mM NaCl, 2 mM TCEP was crystallised in the presence of 48 mM Mg^2+^ and two out of its three substrates, 2 mM IMP and 7.25 mM Asp. PLP was added to the final concentration of 2.42 mM.

Crystallisation trials were carried out at 12 °C in VDXm (Hampton Research) crystallisation plates, or in glass capillaries (Glaskapillaren Mark-Rohrchen fur rontgenographischen Aufnamen, GLAS, W. Muller) of 0.3 mm or 1 mm diameter. Better crystals were obtained in capillaries. The protein complex was introduced into capillaries with the aid of a syringe and hose until it reached the desired height. Thereafter, the crystallisation liquid was added in a ratio of 1:1 or 1:2 (complex to mother liquid). The capillaries were sealed at both ends with wax and were maintained in a horizontal position. The crystallisation solution 85 mM Tris/HCl at pH 8.5, 21% (w/v) PEG 4000, 170 mM Li_2_SO_4_, 15% (v/v) glycerol was used for experiments in capillaries. Good diffracting crystals were also obtained in 85 mM Tris/HCl pH 8.5, 30% PEG 3350 (w/v), 0.5 M (NH_4_)_2_SO_4_ in standard crystallisation plates.

The data sets were collected on SuperNova (Oxford Diffraction/Rigaku) diffractometer with the Cu anode lamp (λ = 1.541838 Å). Crystals were frozen in liquid nitrogen, with glycerol or PEG 3350 as a cryo protectant and data sets from several crystals were collected. Additionally, one data set was also obtained at 4 °C. All data sets collected yielded almost identical structure of the AdSS complex, therefore details of only one structure with the best resolution, as well as R and R_free_ values, obtained from the frozen crystal, is described here and was deposited in the PDB. The data reduction was carried out using the CrysAllis software provided by the diffractometer manufacturer, Oxford Diffraction/Rigaku. To solve the structure, the CCP4 package was used^27^. Molecular replacement technique was employed to address the phase problem by utilising either MolRep^28^ or Phaser^29^ software from the CCP4 suite. Refmac5^30^ and Coot^31^ were used for the refinement of structures. As a model, structure of C-His tag AdSS from *H. pylori* in a complex with IMP (PDB 7PVO) was used^14^. Statistical data describing the refined structure were calculated in Refmac5^30^. The composite omit electron density map was calculated with the Composite Omit Maps program from the Phenix package^32^, with omitted IMP, PLP, sulphate ion and Lys322. Search for potential metal ions in the structure was performed with the CCP4 Coot program, using the command “check highly coordinated water”^33^, and no suspicious blobs of the electron density were found. Additionally, Mg ion was added manually in several blobs of electron density within the active site, and each of them was checked with the CheckMyMetal server (https://cmm.minorlab.org/)^34^ with no positive results.

### Determination of the minimum inhibitory concentration (MIC)

*H. pylori* was cultured at 37 °C under microaerophilic conditions maintained by an atmosphere generator Anoxomat Mark II. Bacteria were incubated for three days on BHI agar plates containing 10% (v/v) FBS and 1% (v/v) *H. pylori* Selective Supplement, followed by overnight growth in the liquid BHI medium supplemented with 10% (v/v) FBS and 1% (v/v) *H. pylori* Selective Supplement.

Inhibition of *H. pylori* by PLP and its metabolic precursors was studied as described previously by Narczyk et al.^13^. Kanamycin 25 μg/mL was used as a positive control^35^, while milli-Q water and BHI medium without bacteria as negative controls. The experiments were carried out in 24-deep-well, flat-bottomed plates, filled in each well with a final volume of 500 μL. Two-fold serial dilutions of inhibitors and an equal volume of inoculated double-strength BHI were added to each well, resulting in an optical density at the start of experiments, OD600, of approximately 0.05 (∼4 × 10^4^ CFU/mL in a final volume). Plates were shaken at 120 rpm under microaerophilic conditions at 37 °C. OD_600_ of samples was measured at the start of the experiment and after 4, 8 and 24 h of incubation^36^. In the negative control consisting of milli-Q water, after 24 h incubation OD_600_ was typically in the range of 1.0–1.5, which corresponded to bacteria concentration of about 10^6^ CFU/mL.

The Christensen test was performed in each of the experiments, according to Knezevic et al.^37^, to ascertain the viability of the *H. pylori* cells. Post-incubation, equal volume of a double strength Christensen’s urea broth was added into each well, and the plates were additionally incubated for 4 h in an aerobic atmosphere at 37 °C. During this time, in wells with viable *H. pylori* urease was produced and converted urea into ammonia and carbon dioxide, changing the pH and the colour of the phenol red indicator present in the medium from orange to purple. All experiments were performed in triplicate.

### Determination of the minimum bactericidal concentration (MBC)

To determine the MBC, after completing a 24 h incubation 10 μL of suspension from each well of the 24-well, flat-bottomed titration plate was withdrawn and dropped on BHI agar plates containing 10% (v/v) FBS and 1% (v/v) *H. pylori* Selective Supplement and incubated under microaerophilic conditions for three days at 37 °C. The minimum bactericidal concentration (MBC) was considered as the lowest concentration at which no growth of bacterial colonies was observed^38^. All experiments were performed in triplicate.

### Cellular uptake of PLP and PI-h by H. pylori

Cellular uptake of PLP and PI-h by *H. pylori* was quantified by measuring the reduction of the inhibitor concentration in the external culture medium after a specific time of incubation^17^^,^^39^. In most cases the absorption detection was used. However, to achieve the best accuracy, the full spectra in the range 200–500 nm were measured to adjust the absorbance at the observation wavelength against the background. The following extinction coefficients were used to calculate the concentration of the tested compounds in the external culture medium: 8200 M^−1 ^cm^−1^ at 318 nm for PI-h and 4900 M^−1 ^cm^−1^ at 388 nm for PLP^19^. For PI-h, LC-MS detection was also used to obtain more accurate results.

The uptake of the tested compound was calculated accordingly:

(3) $$\begin{array}{l} C\text{accumulated }\text{intracellular}=(C\text{inhibitor},\text{8h},37{}^{\circ}C-C(\text{Hp}+\text{inhibitor}),\text{8h},37{}^{\circ}C)\;\\ \;-(C\text{inhibitor},\text{8h},4{}^{\circ}C-C(\text{Hp}+\text{inhibitor}),\text{8h},4{}^{\circ}C) \end{array}$$

where C corresponds to the concentration of the tested compound. Subscripts “inhibitor” and “Hp + inhibitor” refer to the control without bacterial cells, and samples of the *H. pylori* cells incubated in the presence of inhibitor, respectively. Finally, subscript 37 °C and 4 °C refer to two temperature conditions used.

## Results

### Dependence of AdSS inactivation by PLP on inhibitor concentration

**Figure 2.**
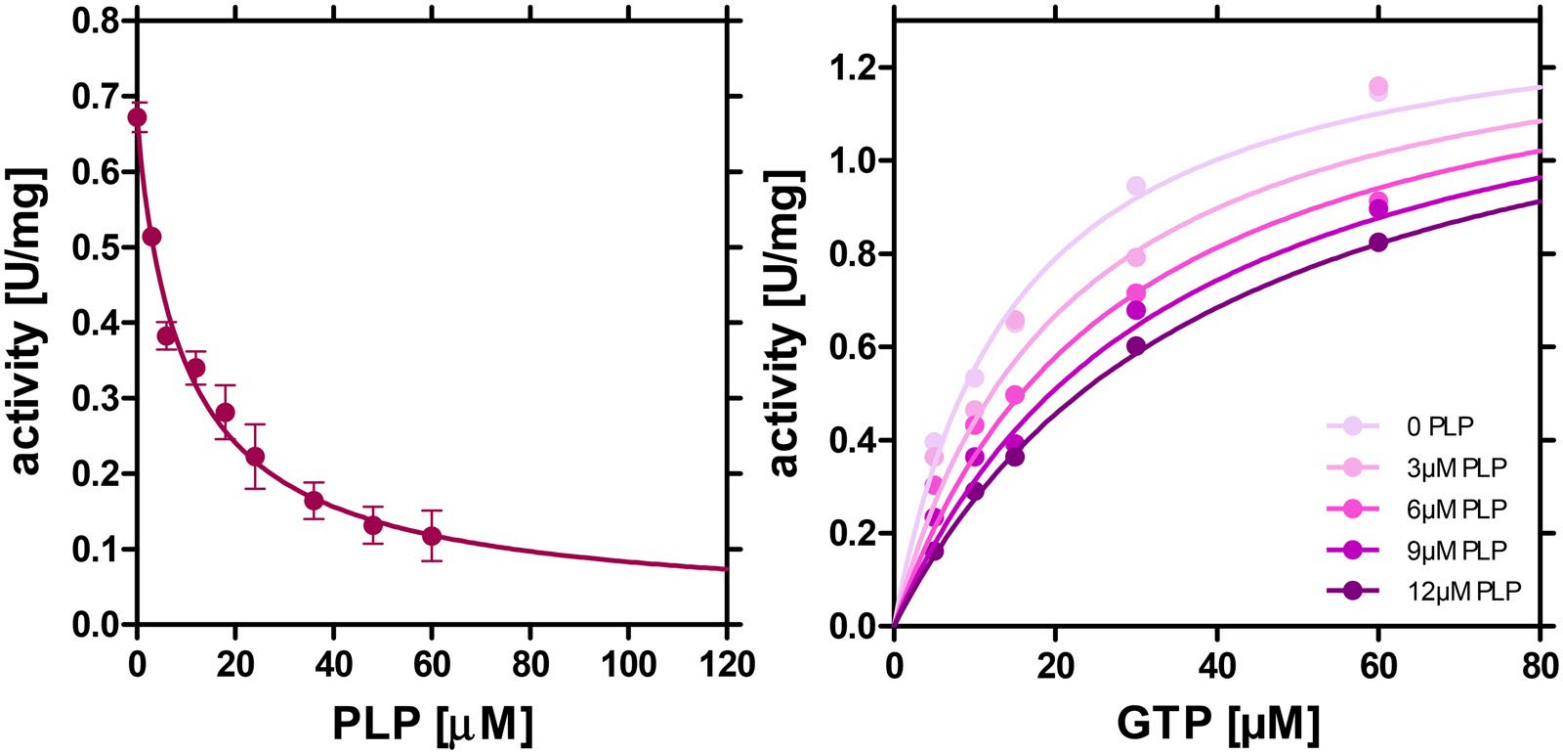
Left panel: Inhibition of *H. pylori* AdSS by PLP at 25 °C in 20 mM Hepes buffer at pH 7.7, 1 mM MgSO_4_, at saturating concentrations of AdSS substrates, IMP (0.15 mM), GTP (0.06 mM) and Asp (5 mM). The four-parameter dose-response curve (Equation (1)) was fitted to the data, yielding IC_50_ = 9.97 (with asymmetric confidence intervals 3.99 to 24.91 µM). Right panel: Inhibition of *H. pylori* AdSS by PLP at 25 °C in 20 mM Tris buffer at pH 7.7, 1 mM MgSO_4_, at saturating concentrations of IMP (0.15 mM) and Asp (5 mM), and variable concentration of GTP. Increasing PLP concentrations, 0, 3, 6, 9 and 12 µM, respectively, are shown by increasing the colour intensity of the points of individual data series. Competitive inhibition model was fitted globally to all data points, and yielded K_i_ = 6.95 ± 0.82 µM.

Inhibition of AdSS from *H. pylori* 26695 strain by PLP was first studied at saturation with all enzyme substrates and increasing concentration of PLP. Data obtained are depicted in Figure 2 (left panel) and revealed that enzyme activity is strongly reduced by the presence of PLP. The four-parameter dose-response curve was fitted to the data, showing that at PLP concentrations higher than ∼60 μM the activity of this enzyme is decreased by 86%, while further increase of the PLP concentration practically inactivates the enzyme (A_eq_ = 0.02 ± 0.09 U/mg). The PLP concentration leading to half of this inhibition was found to be IC_50_ = 9.97 μM (with asymmetric confidence intervals 3.99 to 24.91 μM).

The inhibition model and the inhibition constant were next determined using GTP as variable substrate, and IMP (0.15 mM) and Asp (5 mM) as saturators. This choice was made, as according to Dong and Fromm^18^, PLP competes with this AdSS substrate in the case of the enzyme from *E. coli*. Data for AdSS from *H. pylori* 26695 is shown in Figure 2 (right panel). Competitive inhibition model was globally fitted and found to be appropriate, thus yielding the inhibition constant K_i_ = 6.95 ± 0.82 μM (Table 1). Overall, these findings suggest that PLP inhibits AdSS from the *H. pylori* 26695 strain in a concentration-dependent manner and that this inhibition occurs through competition with GTP.

**Table 1.** Parameters describing formation of the AdSS-PLP complex, reversible and tightly bound (covalently joined due to formation of the Schiff base), and effect of 1 mM vitamin C, acting as a Schiff base reducing agent.

AdSS-PLP complex	Parameter		Standard error or asymmetric confidence intervals	Conditions
reversible	IC_50_	9.97 µM	3.99 to 24.91 µM	Saturation with substrates
	A_∞_	0.02 U/mg	±0.09 U/mg	Saturation with substrates and PLP
	K_i_	6.95 µM	±0.82 µM	Competition with GTP
Schiff base bound	IC_50_^eq^	0.028 µM	0.011 to 0.068 µM	In equilibrium, Saturation with IMP, Asp and PLP, no GTP
	A_∞_	0.08 U/mg	±0.03 U/mg	
	k	0.73 min^-1^	±0.09 min^-1^	
+ 1 mM Vitamin C	A_∞_	0.03 U/mg	±0.02 U/mg	PLP 100 µM, incubated
	k	14.47 min^-1^	±1.89 min^-1^	with AdSS, no substrates present
No vitamin C	A_∞_	0.12 U/mg	±0.01 U/mg	
	k	0.037 min^-1^	±0.002 min^-1^	

Similar experiments were done with pyridoxal, while surprisingly revealing that this metabolic precursor of PLP does not inhibit *H. pylori* AdSS, even at concentrations as high as 400 μM (data not shown). This finding highlights the significance of the phosphate group of PLP, lacked in pyridoxal (see Figure 1), in mediating interactions between PLP and AdSS.

### Time dependence of AdSS inactivation by PLP

**Figure 3.**
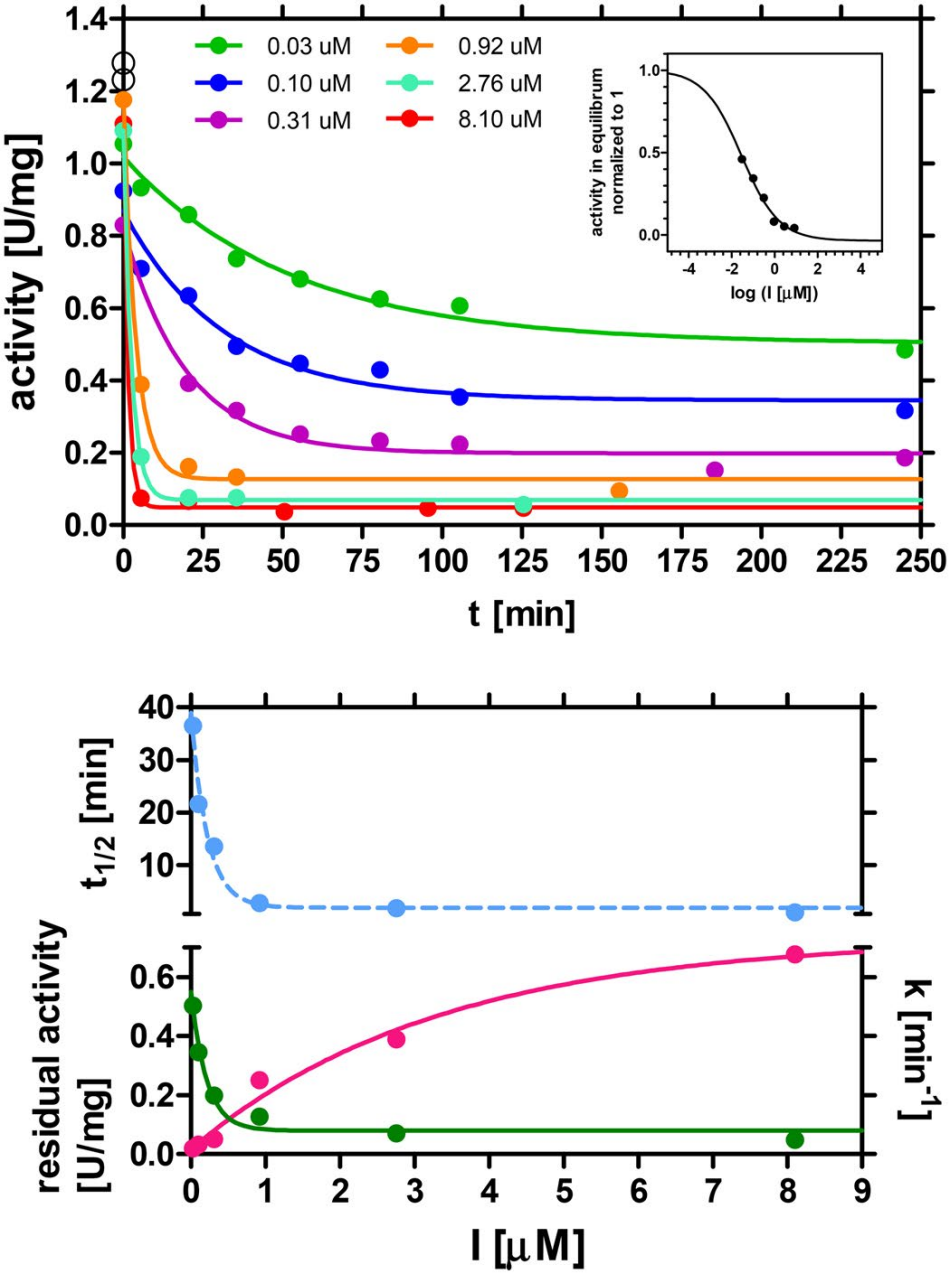
Upper panel: Time dependence of inactivation of *H. pylori* AdSS by PLP present in various concentrations. Enzyme (22 nM) was incubated at 25 °C in 20 mM Hepes buffer at pH 7.7, with various concentrations of PLP: 0.03 µM (green), 0.1 µM (blue), 0.3 (violet), 0.92 µM (orange), 2.76 µM (aquamarine), 8.1 µM (red). Activity at time 0 and no PLP present is shown by the black open circles. One-phase exponential decay (Equation (2)) was fitted to each of the traces to get for each of the PLP concentrations the rate constant, k, of the inactivation process and the residual enzyme activity when equilibrium is reached, A_eq_, and calculate half-time of the inactivation process t_1/2_. The inset shows activity in equilibrium vs. inhibitor concentration, with the four-parameter dose-response curve (Equation (1)) fitted, which yields IC_50_^eq^ = 0.028 µM (with asymmetrical error range from 0.011 to 0.068 µM). Lower panel: Dependence of the rate constant, k, residual enzyme activity, A_eq_, and half-time of the inactivation process, t_1/2_, on the inhibitor, PLP, concentration [I]. At saturation with the inhibitor these parameters are: k = 0.73 ± 0.09 min^−1^ (magenta); activity A_eq_ = 0.08 ± 0.03 U/mg (green) and t_1/2_ = 0.95 ± 0.11 min (blue).

To determine how inactivation of AdSS caused by PLP proceeds with time, the enzyme at low concentration (0.022 μM) was incubated at 25 °C with PLP at various concentrations (ranging from 0.03 μM to 8.1 μM). The mixture contained all components necessary for the reaction, except GTP, as it was shown above that PLP competes with this substrate. Aliquots of 900 μL of this mixture were withdrawn at specific time intervals, placed in the cuvette and 100 μL of 0.6 mM GTP was added to initiate the reaction and follow its progress. The results obtained are shown in Figure 3.

As shown in Figure 3 (upper panel), in conditions without the presence of GTP, where PLP could interact with the enzyme, even a low concentration of PLP (0.03 μM) causes a substantial reduction (∼50%) of the enzyme activity compared to the control (after 4 h of incubation being 1.23 U/mg vs. 0.48 U/mg, respectively). An increase in PLP concentration results in a rapid decrease in the enzyme activity. For example, when PLP is present at concentration of 8.1 μM the enzyme activity decreased to only 0.04 U/mg after approximately 10 min (reduction by ∼97%). To further analyse the inactivation process, rate constants of the inactivation process (k) were determined at various PLP concentrations. The results, as shown in the lower panel of Figure 3, displayed a saturation curve that reached a maximum at k = 0.73 ± 0.09 min^−1^. This value corresponds to half-time (t_1/2_) of the inactivation process, which was estimated to be 0.95 ± 0.11 min. The half-time of the inactivation process was calculated for each PLP concentration and is depicted in the same figure.

The data obtained show that PLP is a potent, slow-binding and tight-binding inhibitor of AdSS from *H. pylori*. Our experiments demonstrate that even at a concentration of 1 µM, PLP causes almost complete inactivation of the enzyme (by 90%) which increases to 97% at a concentration of 8.1 µM. Moreover, this inactivation occurs within a few minutes. This behaviour of the PLP-AdSS reaction according to Dong and Fromm^18^ is akin to that of the enzyme catalysis as it proceeds in two steps. In the first step the rapid formation of the noncovalent enzyme-PLP complex is observed. Subsequently, in the second step, the Schiff base is formed between PLP and AdSS leading to inactivation of the enzyme.

The inset of Figure 3 shows activity in equilibrium vs. inhibitor concentration, with the four-parameter dose-response curve, Equation (1), fitted, which yields IC_50_^eq^ = 0.028 μM (with asymmetrical error range from 0.011 to 0.068 μM).

### Reduction of the Schiff base by vitamin C

The Schiff base formation is a reversible process. Only further reduction of the C = N bond leads to the formation of the irreversible complex (Figure 1S). Reduction in the study of Dong and Fromm^18^ was achieved by adding sodium borohydride, while Torabizadeh et al.^40^ utilised vitamin C to obtain reduction in their study on endoinulinase. Given the potential medical applications, we decided to investigate the efficacy of vitamin C as a reducing agent, considering its promising properties.

**Figure 4.**
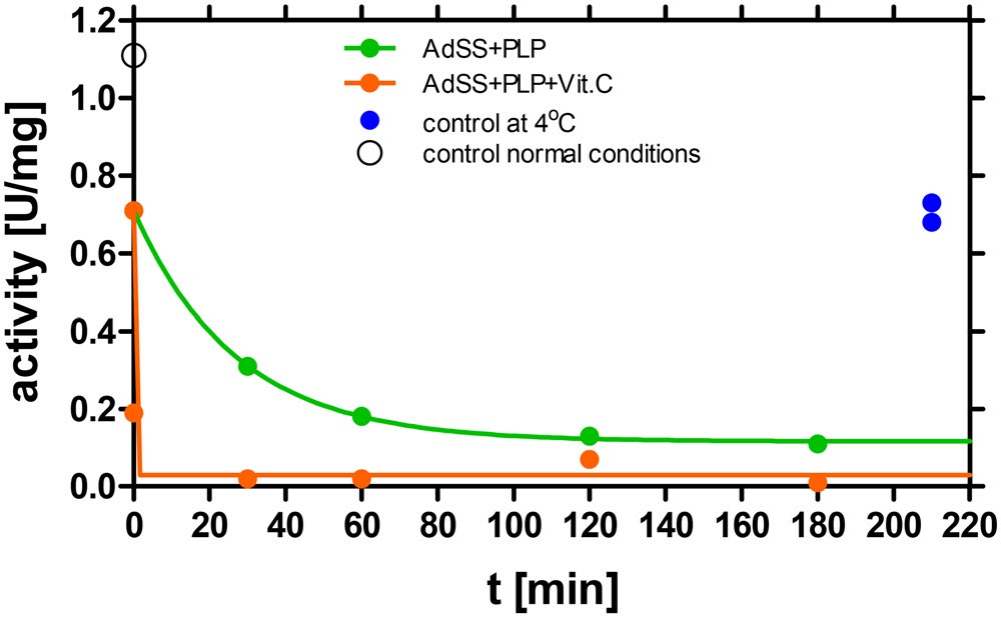
Reduction by vitamin C of the Schiff base formed between AdSS and PLP. The enzyme, 4.15 µM, was incubated at 25 °C in 20 mM Hepes buffer at pH 7.7 with 100 µM PLP in the absence (green) and in the presence (orange) of 1 mM vitamin C. Enzyme activity assayed in the standard conditions, observed at the start of the experiment prior to addition of PLP and vitamin C, and at the end of the experiment for the sample without PLP and vitamin C kept in ice, is marked by a black open circle and a blue dots, respectively. In the presence of 1 mM of vitamin C the rate constant k increases from k = 0.037 ± 0.002 min^−1^ to k = 14.47 ± 1.86 min^−1^, while the residual enzyme activity A_eq_, drops from A_eq_ = 0.12 ± 0.01 U/mg to practically zero, A_eq_ = 0.03 ± 0.02 U/mg.

The AdSS enzyme, at the concentration of 4.15 μM, was incubated at 25 °C in 20 mM Hepes/NaOH buffer pH 7.7 with 100 μM PLP in the absence and in the presence of 1 mM vitamin C. Data obtained is presented in Figure 4 and indicate that vitamin C plays a crucial role in accelerating the formation and stabilisation of the AdSS-PLP complex. This is evident from the complete AdSS inactivation observed under such conditions (the residual activity, A_eq_ = 0.03 ± 0.02 U/mg), and the rate constant of the complex formation, k = 14.47 ± 1.86 min^−1^, corresponding to half-time of inactivation t_1/2_ = 0.048 ± 0.097 min. On the other hand, in the same conditions without vitamin C the residual enzyme activity is observed (A_eq_ = 0.12 ± 0.01 U/mg; ∼10% of the starting activity) and the inactivation process is nearly 400 times slower (k = 0.037 ± 0.002 min^−1^ and t_1/2_ = 18.67 ± 0.68 min (Table 1)). This is the expected effect caused by the reduction of the Schiff base, which leads to the formation of the irreversible AdSS-PLP complex and thus to complete inactivation of the enzyme. Moreover, the inactivation proceeds more quickly in the presence of vitamin C, which is consistent with the decrease of the Schiff base complex concentration due to its further reduction.

Reduction of the Schiff base AdSS-PLP complex is also observed when vitamin C is added after AdSS-PLP reversible complex is already formed (data not shown).

### Three-dimensional structure of AdSS complex with PLP

In order to characterise in detail the interactions of PLP with AdSS from *H. pylori,* we have determined the three-dimensional structure of the enzyme-ligand complex by the X-ray diffraction.

C-His tag AdSS was complexed with two out of its three substrates (IMP and Asp) and with PLP, in the presence of Mg^2+^. GTP was omitted, as according to the studies described above it competes with PLP, hence we expect to find PLP in the GTP-binding site. Several crystallisation settings and crystallisation conditions, described in the Materials and Methods section, gave well diffracting crystals. All structures obtained are almost identical, hence just one with the best resolution, as well as R and R_free_ values, is described here. The data collection and refinement parameters for the best data set are summarised in Table 2.

**Table 2.** Diffraction data, its statistical analysis, and parameters of the AdSS structure deposited in the PDB.

Structure	*H. pylori* AdSS with PLP and IMP PDB 8QWA
Resolution range (Å)	29.80–1.85 (1.89–1.85)^a^
Space group	I 1 2 1
Unit cell: *a, b, c* (Å) *α, β, γ* (°)	68.9 61.1 119.3 90.0 98.9 90.0
Total number of reflections	984 730 (32 859)
Number of unique reflections	41 958 (2580)
Multiplicity	23.5 (12.7)
Completeness (%)	100.0 (100.0)
Mean *I*/σ(*I*)	48.0 (8.3)
Wilson B factor	12.36
*R_merge_*	0.049 (0.251)
*R_meas_*	0.05 (0.261)
*R_pim_*	0.01 (0.073)
Reflections used for refinement	41 870
Reflections used for calculation of *R_free_*	4135
*R_work_*	0.166
*R_free_*	0.201
Atoms without hydrogen atoms for:	
Macromolecules	3199
Ligands	49
Solvent	267
Number of amino acids	402
RMS(bonds)	0.0103
RMS (angles)	1.759
Ramachandran optimal	384 (95.52%)
Ramachandran allowed	16 (3.98%)
Ramachandran outliers	2 (1.43%)
Rotamer outliers (%)	13
Atomic overlapping index	4.6
Average B-factor for:	
Macromolecules	19.29
Ligands	29.56
Solvent	26.7

^a^Statistical data for the highest resolution shell are given in parenthesis.

Our findings reveal that the AdSS-ligand complex crystallises in the I 1 2 1 space group, with a monoclinic unit cell of dimensions 69.9 Å, 62.1 Å, 119.3 Å, 90.0°, 98.9°, 90.0°. The asymmetric unit contains one monomer of the protein (thus half of the functionally capable enzyme). The overall enzyme structure is similar to the structures of this enzyme obtained previously^14^, namely in binary complex with IMP (RMSD 0.22, as calculated by the PDB Pairwise Structure Alignment) and to the fully ligated enzyme structure, with IMP-6-phosphate, hadacidin, GDP and magnesium ion (RMSD 0.62, as calculated by the PDB Pairwise Structure Alignment). The core of the enzyme comprises a β-sheet composed of 9 β-strands (8 mutually parallel and one anti-parallel), flanked by more than 10 α-helices of various length. The shallow but very elongated active site pockets are positioned on the opposite sides of the monomer-monomer interface. However, the long side chain of Arg135 from the neighbouring subunit extends towards the active site of the paired monomer and participates in the ligand binding, namely in binding of IMP, thus indicating the necessity of a dimeric architecture for the formation of the catalytically active AdSS. The affinity C-His tag comprising 6 histidine residues is not visible in the electron density suggesting its high flexibility. The C-terminal end of the protein is positioned at the opposite side of the active site, making it not likely that the tag would affect the enzymatic properties of *H. pylori* AdSS. This is consistent with the observed similar kinetic properties of the WT and C-His tag enzyme variants^14^.

**Figure 5.**
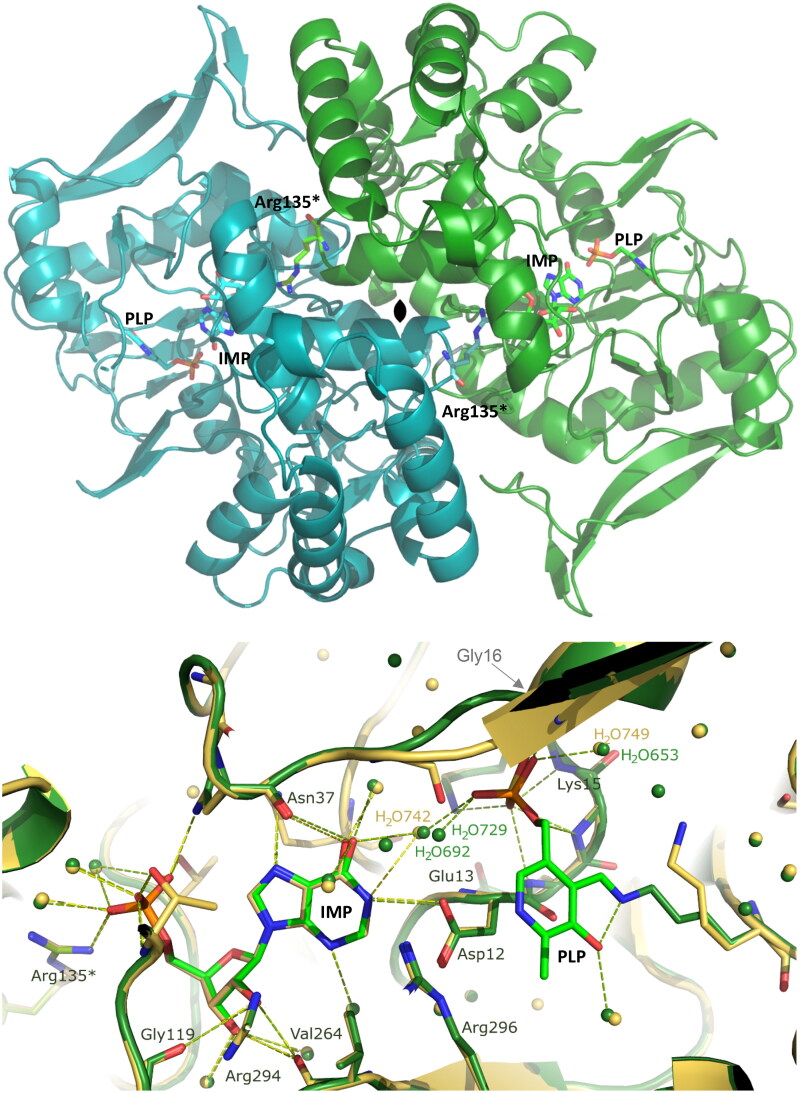
Upper panel: The overall structure of the *H. pylori* AdSS dimer, which is formed by two identical monomers (shown in green and turquoise) as the result of a two-fold symmetry axis (denoted in the centre). The active sites are located on the opposite sides of the monomer-monomer interface and have the form of elongated clefts lying near the surface of the protein. The side-chain of amino acid Arg135 from each monomer (marked with an asterisk) completes the active site of the neighbouring subunit. The C-terminus in each monomer, to which the histidine tag is attached but not visible in the electron density, is positioned on the opposite side of the protein compared to the active site. Lower panel: Active site of the AdSS ternary complex with PLP and IMP (green, 8QWA) overlaid with the active site of the binary complex of the enzyme with only IMP (yellow, 7PVO) ^14^. Hydrogen bond network observed in both complexes is shown with dashed lines, green and yellow, respectively. Water molecules are represented by green and yellow spheres (the numbers of some of them are shown and correspond to their numbers in the respective PDB files). Gly16, which is here covered by the beta sheet of the structure, and which interacts through the main chain N atom via a hydrogen bond with the phosphate group of PLP (see Table 3), is shown by the arrow. Arg135 from the adjacent subunit (marked with an asterisk) is seen to be involved in IMP binding at the active site located in the neighbouring monomer of the dimer.

**Figure 6.**
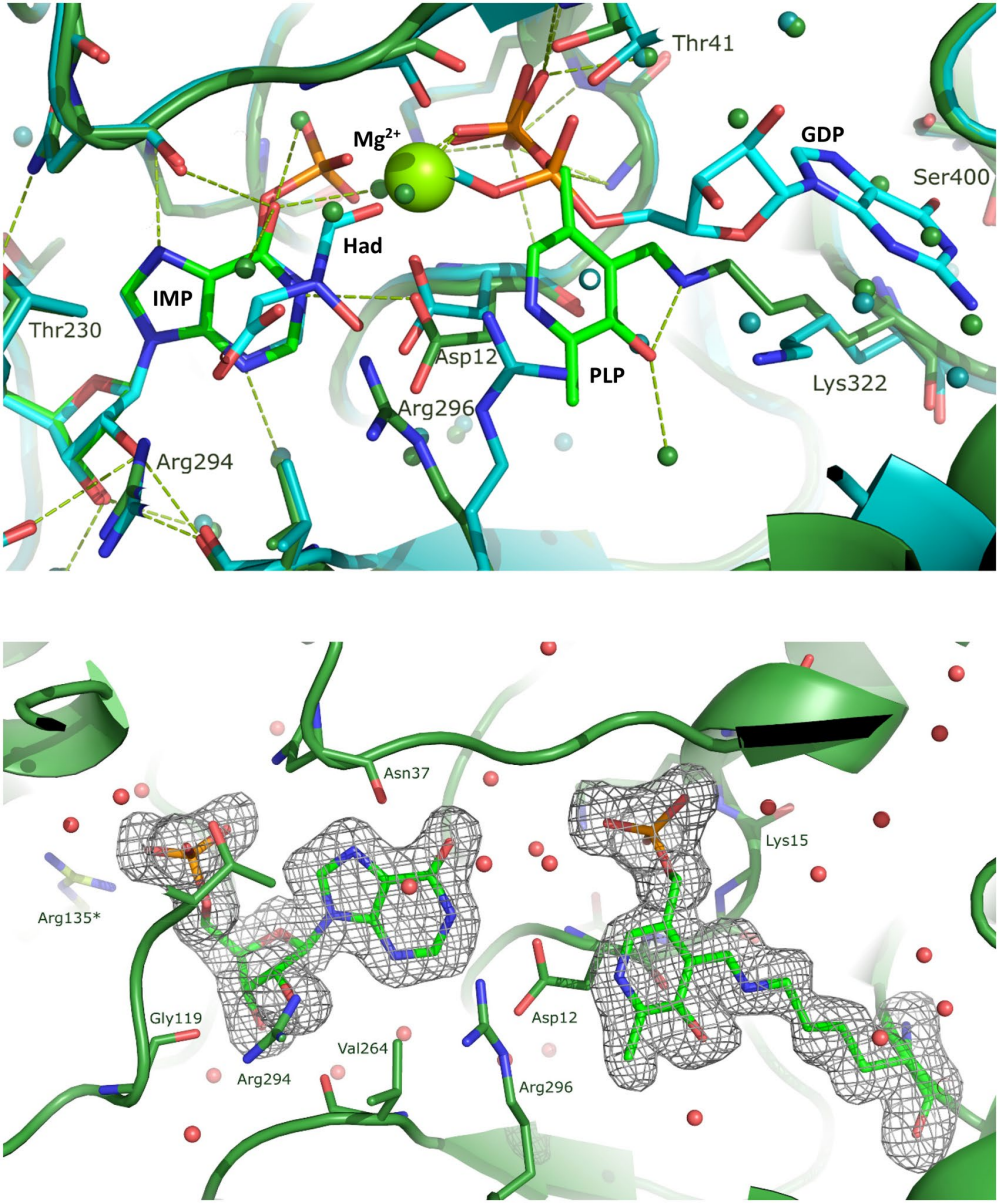
Upper panel: Active site of the AdSS ternary complex with PLP and IMP (green) overlaid with the active site of the AdSS complex with 6-phosphoryl-IMP (IMO), hadacidin (Had), GDP and Mg^2+^ (cyan, 6ZXQ) ^14^. Hydrogen bond network observed in both complexes are shown with dashed green and cyan lines, respectively. Water molecules are represented by spheres (green and cyan, respectively). Mg^2+^ ion is represented by larger light green sphere. Lower panel: The composite omit electron density map for the ligands in the active site of the AdSS complex with PLP and IMP. The Fo-Fc map is shown, contoured at 3.0 σ level.

Ligands bound in the active site are IMP and PLP (Figures 5 and 6), even though before crystallisation AdSS was preincubated with four potential ligands: IMP, Asp, PLP and Mg^2+^. All of the hydrogen bond contacts, together with their lengths, observed in the enzyme active site, are listed in Table 3. IMP is well visible in the electron density, and its position in the active site is the same as in the structure of the binary complex of *H. pylori* AdSS and IMP solved by our group previously (7PVO)^14^. Within a hydrogen bonding distance with IMP there are the following amino acids: Asp12, Asn37, Gly119, Thr121, Thr230, Val264, Arg294, and also Arg135 from the neighbouring subunit (Table 3 and Figure 5).

**Table 3.** Contacts, putative hydrogen bonds and one covalent bond, observed in the active site of *H. pylori* AdSS between the enzyme and three ligands present, PLP, IMP and sulphate ion.

PLP		IMP		SO_4_	
Hydrogen bond	Length [Å]	Hydrogen bond	Length [Å]	Hydrogen bond	Length [Å]
O3 ··· Nζ Lys322	2.41	O3P ··· NH1 Arg135*	2.80	O1 ··· NH1 Arg296	3.08
O3 ··· O H_2_O634	2.60	O3P ··· O H_2_O657	2.65	O2 ··· O H_2_O722	2.78
C4A ― Nζ Lys322	1.26	O3P ··· O H_2_O6761	2.69	O2 ··· O H_2_O777	3.18
O4P ··· N Gly14	3.39	O2P ··· Oγ1 Thr230	2.61	O3 ··· NH_2_ Arg294	3.10
O1P ··· N Gly16	2.95	O2P ··· Nδ2 Asn37	2.79	O3 ··· O H_2_O723	2.78
O1P ··· N Lys15	3.30	O1P ··· Oγ1 Thr121	2.56	O4 ··· Nζ Arg294	2.69
O1P ··· O H_2_O653	2.64	O1P ··· N Thr121	2.83	O4 ··· Nε Arg294	3.34
O2P ··· Nζ Lys15	2.61	O1P ··· O H_2_O683	2.77		
O2P ··· N Lys15	2.86	O3’ ··· O Val264	2.99		
O2P ··· N Gly14	2.97	O3’ ··· O H_2_O631	2.59		
O3P ··· O H_2_O692	2.72	O3’ ··· O H_2_O713	3.10		
O3P ··· O H_2_O729	2.86	O2’ ··· O Val264	2.73		
		O2’ ··· NH_2_ Arg294	2.79		
		O2’ ··· O Gly119	3.21		
		N3 ··· O H_2_O662	2.96		
		N1 ··· Oδ1 Asp12	3.06		
		N1 ··· O H2O723	3.30		
		O6 ··· O H2O692	3.01		
		O6 ··· O Asn37	3.37		

··· - hydrogen bond, ― - covalent bond, * - amino acid from the neighbouring subunit. Water molecules are numbered according to the PDB file 8QWA, in which the structure is deposited.

PLP indeed occupies the part of the active site where GTP or GDP bind, which can be noticed when the structure solved in this study is compared with the structure of the *H. pylori* AdSS complex with 6-phosphoryl-IMP (IMO), hadacidin, GDP and Mg^2+^ (6ZXQ)^14^ (Figure 6). This observation is in line with the results of kinetic studies that point to competition of these two ligands, namely GTP and PLP, for *H. pylori* AdSS. Additionally it correlates well with results obtained previously by Dong and Fromm^18^ in the case of *E. coli* AdSS. Phosphate group of PLP is clearly visible in the electron density. It is located in the position of β phosphate group of GTP (GDP), and it maintains the hydrogen bond network of this phosphate group, namely forms hydrogen bonds with backbone atoms of Lys15 and Gly14, and with Nζ of Lys15 (Table 3 and Figure 2S). The electron density map indicates that the aromatic ring of pyridoxal 5′-phosphate (PLP) is somewhat less distinct, yet the position of PLP within the active site is unequivocal, as we have determined through multiple structural investigations of this complex (refer to the Materials and methods section “*Crystallization of AdSS-PLP complex and diffraction studies”*). The aromatic ring of PLP is bound covalently to the side chain atom Nζ of Lys322 utilising for this purpose the aldehyde group of PLP (see Figure 5). The distance between C4A atom of PLP and Nζ atom of the Lys322 is 1.26 Å, thus confirming formation of the covalent bond formation. This finding is consistent with the time dependence observed in the inhibition of AdSS by PLP, as the initially formed reversible inhibitor-enzyme complex undergoes slow conversion to the Schiff base, in which PLP is covalently bound to AdSS. It is worth comparing in more detail the positions of PLP and GDP observed in the presently solved structure and that of the *H. pylori* AdSS complex with IMO, hadacidin, GDP and Mg^2+^. It indicates that the place of guanosine of the GDP (GTP) nucleotide in the structure with PLP is occupied by the extended side chain of the Lys322, while the covalently bound aromatic ring of PLP is located more deeply in the active site pocket and approaches the place of the α phosphate group of GTP (GDP), however it does not form hydrogen bond with the enzyme (Table 3, Figure 6 and Figure 2S). It shows that the anchor of the PLP molecule in the AdSS active site, except for the covalent bond with Lys322, is a phosphate group. The anchoring of the PLP molecule using a phosphate group is probably a prerequisite for the subsequent formation of a Schiff base between PLP and AdSS. This observation explains why pyridoxal, which lacks a phosphate group, has no affinity for *H. pylori* AdSS.

### Inhibition of H. pylori wild-type and clinical strains by PLP and its metabolic precursor - pyridoxal

**Figure 7.**
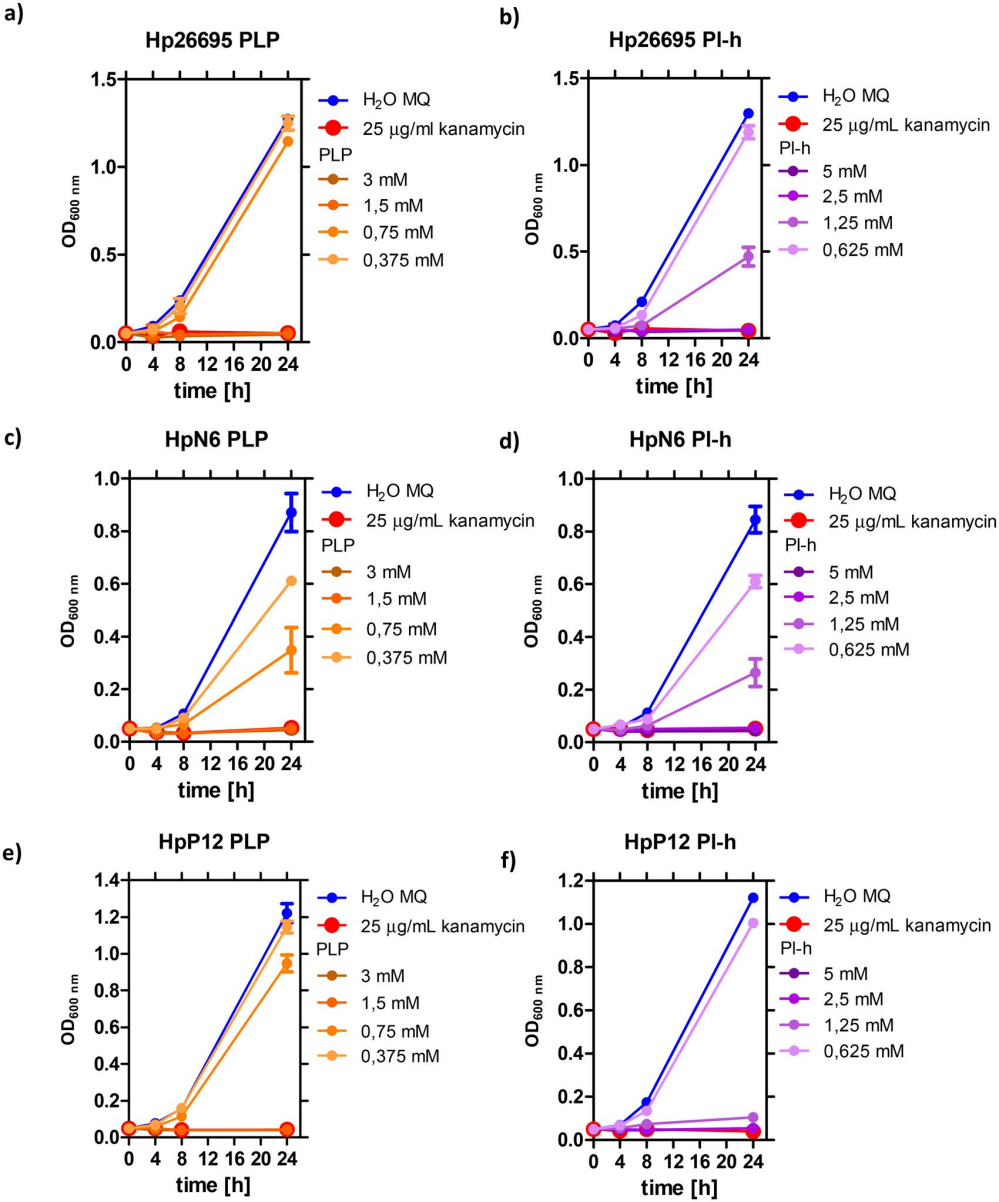
Growth curves of *H. pylori* 26695 strain (a,b), N6 strain (c,d) and P12 strain (e,f), in the presence of various concentrations of PLP and PI-h. MIC values determined from these curves are shown in Table 4 and were also confirmed by the Christensen urease test as described in Materials and Methods section and shown on Figure 9 for the pair PI-h/strain 26695 as an example.

Noteworthy, PLP has been found to exhibit slow-binding and tight-binding inhibitory properties against AdSS from *H. pylori*. According to Liechti and Goldberg^11^, this enzyme is essential for survival of *H. pylori*. Therefore, in the next step, we have determined effects of PLP and its metabolic precursors on the replication of the three *H. pylori* wild-type strains (26695, N6 and P12). The tested compounds included PLP, PI-h, pyridoxine, pyridoxine in the form of hydrochloride, and pyridoxamine in the form of dihydrochloride. Results show that except for PLP and PI-h, these compounds do not significantly inhibit bacterial growth even up to 10 mM concentration. In contrast, PLP and PI-h are effective in supressing the growth of all three tested wild-type *H. pylori* strains (Figure 7) with similar MIC values of 370 µg/ml (1.5 mM) for PLP and 509 µg/ml (2.5 mM) for PI-h (Table 4).

**Table 4.** Minimum inhibitory concentration (MIC) and minimal bactericidal concentration (MBC) values of PLP and PI-h for wild-type and metronidazole (MTZ) and/or clarithromycin (CLR) resistant *H. pylori* strains.

*H. pylori* strain	Resistant to:	MIC (MBC) PLP		MIC (MBC) PI-h	
		[mM]	[µg/mL]	[mM]	[µg/mL]
26695	–	1.5 (3.0)	370 (740)	2.5 (2.5)	509 (509)
N6	–	1.5	370	2.5	509
P12	–	1.5	370	2.5	509
M92	CLR	≥0.75; ≤1.5	≥185; ≤370	1.5	509
M93	MTZ	0.75	185	1.25	255
M26	MTZ + CLR	0.75	185	1.25	255
M91	MTZ + CLR	≥0.75; ≤1.5	≥309; ≤370	1.25	255

**Figure 8.**
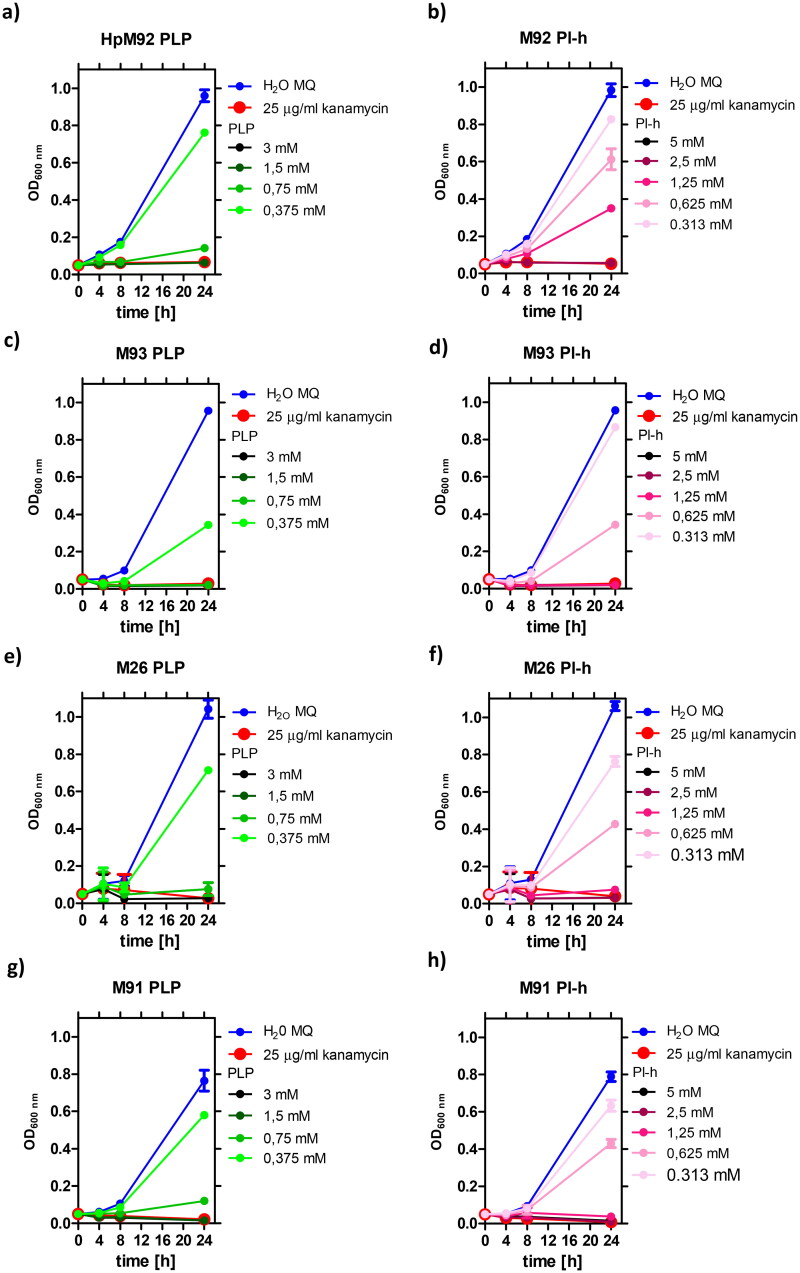
Growth curves of *H. pylori* strain M92 resistant to clarithromycin (a,b), M93 resistant to metronidazole (c,d), and M26 (e,f) and M91 (g,h) resistant to both drugs, in the presence of various concentrations of PLP and PI-h. MIC values determined from these curves are shown in Table 4 and were also confirmed by the Christensen urease test as described in Materials and Methods section.

Additionally, we studied the effect of PLP and PI-h on four clinical antibiotic-resistant *H. pylori* strains: M92 (resistant to clarithromycin), M93 (resistant to metronidazole), as well as M26 and M91 (resistant to both drugs). The results for all tested scenarios are shown in Figure 8, while the MIC values are summarised in Table 4. In general, the tested antibiotic-resistant *H. pylori* strains present greater sensitivity to PLP and its precursor (PI-h) compared to the wild-type *H. pylori* strains. Exception is M92 strain, which has a MIC value of PI-h in a similar range as three tested wild-type strains (509 µg/mL, 2.5 mM). For three of the resistant strains (M26, M91 and M93), the MIC values for both compounds are half as low as those for the wild-type strains, being 185 µg/mL (0.75 mM) for PLP and 255 µg/mL (1.25 mM) for PI-h. For other two antibiotic-resistant strains (M91 and M92), the MIC values for PLP ranged from 185 µg/mL (0.75 mM) to 370 µg/mL (1.5 mM) as indicated in Table 4.

**Figure 9.**
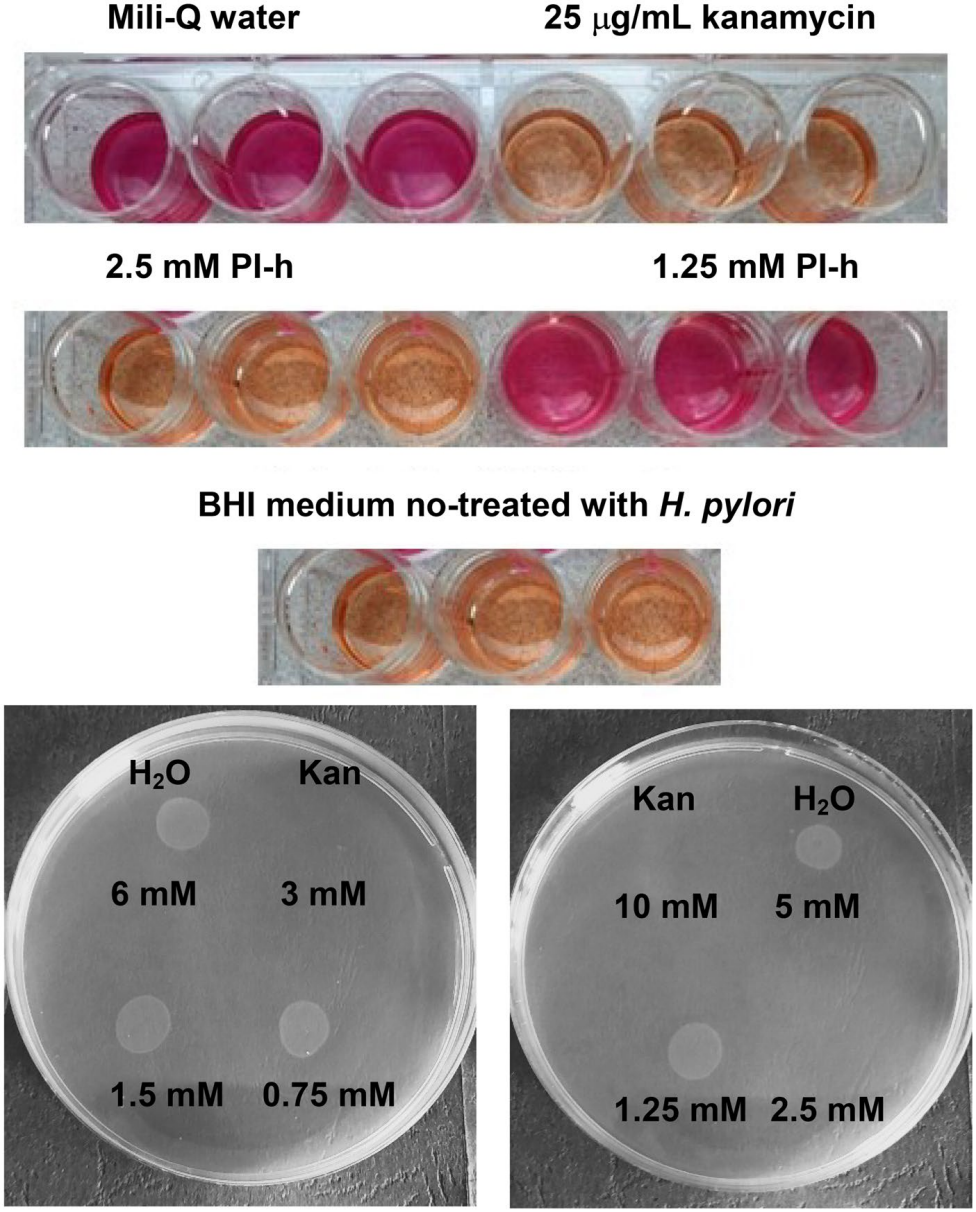
Upper panel: example of the Christensen urease test, here for the pair PI-h/*H. pylori* strain 26695, confirming MIC = 2.5 mM for this pair obtained from the growth curves experiment (Figure 7 panel b). Kanamycin, 25 µg/mL served as a positive control, while water and BHI medium not treated with *H. pylori*, as negative controls. Lower panel: example of the MBC determining experiment, here for the PLP (left) and Pl-h (right) against *H. pylori* strain 26695, showing that MBC is 3 mM and 2.5 mM, respectively. Kan indicates a positive control (25 µg/ml of kanamycin).

In addition to the MIC values, the minimum bactericidal concentrations (MBC) were determined for most of the tested strains (Figure 9). When testing the *H. pylori* 26695 strain, the MBC for PI-h and PLP was equal to MIC and 2 × MIC, respectively. Similar results were obtained for other strains (Table 4). Strains M26 and M92 exhibit MBC values for both compounds twice as high as the MIC, counting 740 µg/mL and 1018 µg/mL for PLP and Pl-h, respectively. Another two clinical strains, M91 and M93, display the MBC of PLP on the same level as their MIC (370 µg/mL), while for PI-h the MBC reaches the value of 4 × MIC (1018 µg/mL). All parameters are presented in Table 4.

We demonstrated that the presence of vitamin C effectively stabilises the AdSS-PLP complex *in vitro*. Following this finding, we sought to explore the potential antibacterial synergy between PLP and vitamin C against the *H. pylori* 26695 strain. For this reason, we used a sub-minimal inhibitory concentration (sub-MIC) of 0.6 mM of PLP in our experiments. Our results indicate that vitamin C concentrations up to 10 mM combined with 0.6 mM of PLP had no discernible effect on *H. pylori* proliferation (data not shown). However, together with the increase in the vitamin C concentration to 12 mM, a slight inhibition of bacterial growth was observed (∼20%; Figure 3S).

### Cellular uptake of PLP and PI-h by H. pylori 26695 strain

**Figure 10.**
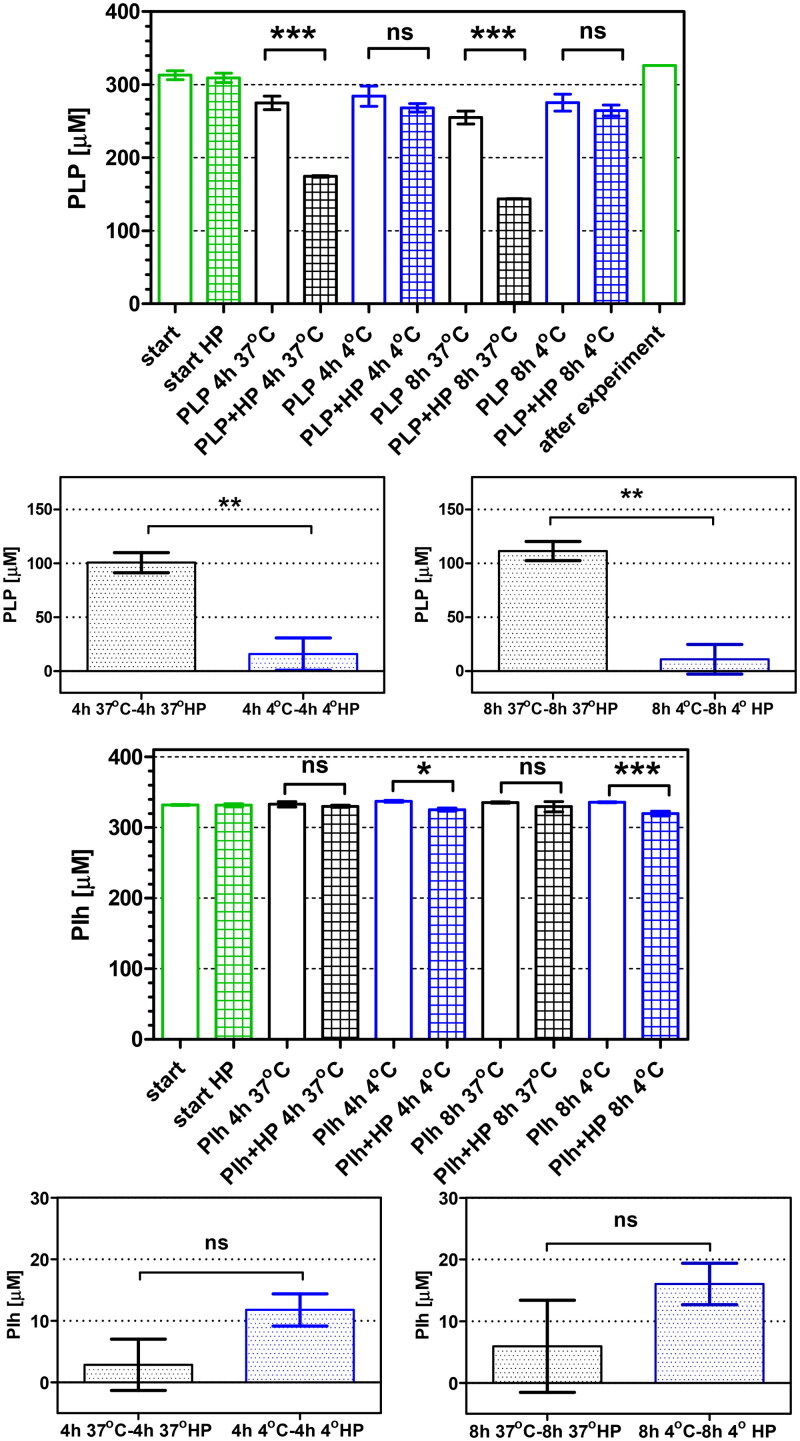
Cellular uptake of PLP and Pi-h by the *H. pylori* 26695 strain after 4 h and 8 h of incubation, quantified by UV absorbance detection. Green bars show inhibitor concentration observed at the start of the incubation. Black and blue bars show data (extracellular inhibitor concentration) obtained for incubation at 37 °C and 4 °C, respectively. Open bars indicate inhibitor incubated in the F12 medium, while filled bars indicate inhibitor incubated in F12 medium containing *H. pylori* cells. Results are mean ± SEM, *n* = 3, **** *p* < 0.0001; *** *p* < 0.001; ** *p* < 0.01, * *p* < 0.05.

In order to investigate the phenomenon conditioning differences between concentrations of the tested compounds effectively inhibiting the native enzyme (AdSS from *H. pylori*) and bacterial growth, we examined the cellular uptake of PLP and PI-h by the *H. pylori* 26695 strain. The previously developed method was utilised^17^^,^^39^ to measure the reduction of inhibitor concentration in the external culture medium. The results are depicted in Figure 10 and in Table 5, and demonstrate that PLP effectively penetrates cells of the *H. pylori* 26695 strain. The initial external concentration of PLP (390 µM) was reduced by 84.7 ± 17.6 µM and 100.4 ± 16.3 µM after 4 h and 8 h incubation, respectively. In contrast, when the *H. pylori* 26695 strain was incubated with PI-h, the initial concentration of 325 µM remained practically unchanged. To confirm the lack of penetration of PI-h into bacterial cells, we conducted experiments with lower initial concentration of 35.4 µM. To improve the detection method, we applied here also the LC-MS, which is expected to be in principle more accurate than the UV absorbance method^17^. In line with this, the UV absorbance detection method showed no penetration of the compound after 4 h and 8 h incubation (Table 5). Although after 4 h of experiment LC-MS show similar results, a difference was noticed after 8 h. Here, LC-MS detected decrease in the external concentration of PI-h by ∼10% (3.58 ± 0.55 µM; Table 5).

**Table 5.** Quantification of the cellular uptake of PLP and PI-h by *H. pylori* 26695 strain cells.

Compound	Starting inhibitor concentration [µM]	Method	Observation wavelength [nm]	Intracellular accumulation [µM]	t-test
PLP	390	UV absorption	388	84.7 ± 17.6 after 4 h 100.4 ± 16.3 after 8 h	****
PI-h	325	UV absorption	318	−8.9 ± 4.9 after 4 h −10.1 ± 8.2 after 8 h	ns
	35.4	UV absorption	318	−1.88 ± 0.77 after 4 h 0.80 ± 0.53 after 8 h	ns
					ns
	35.4	LC-MS	–	0.10 ± 0.84 after 4 h 3.58 ± 0.55 after 8 h	ns ***

*Notes:* The extracellular inhibitor concentration was determined by UV absorption spectra of the tested compounds and LC-MS detection methods ^17^. The t-test was used to check for a significant difference in the extracellular inhibitor concentration between supernatants incubated with and without *H. pylori* cells. The intracellular accumulation of each compound was determined using Equation (3) as described in the text. Results of the t-test are marked as follows: **** *p* < 0.0001; *** *p* < 0.001; ns *p* > 0.05, no significant difference.

## Discussion

Vitamin B6 is a cofactor for nearly 160 enzymatic reactions in the cell. First of all, it participates in the biosynthesis and breakdown of amino acids, fatty acid biosynthesis and glycogen breakdown^41^^,^^42^. Yeast and bacteria can synthesise vitamin B6 *de novo*^43^. Grubman et al.^44^ discovered in the *H. pylori* genome homologs of the *pdxA* and *pdxJ* genes found in *E. coli*, encoding enzymes of *de novo* PLP biosynthesis. The same paper shows that vitamin B6 in addition to fulfilling its basic metabolic functions, is necessary in *H. pylori* for the synthesis of flagella enabling the movement of bacteria, as well as for chronic colonisation of the mouse stomach. Therefore, inhibition of *de novo* synthesis of vitamin B6 in *H. pylori* may constitute a very promising therapy targeting virulence of this pathogen^44^.

In line with this promising data, our study demonstrates the potent inhibitory effect of pyridoxal 5′-phosphate (PLP), the active form of vitamin B6, on AdSS from *H. pylori.* Initially PLP forms a reversible complex with the enzyme with the IC_50_ of 9.97 µM (with asymmetric confidence intervals from 3.99 to 24.91 µM), which over time converts into a tight-binding complex with the IC_50_^eq^ of 0.028 µM (with asymmetric confidence intervals from 0.011 to 0.068 µM). This transformation is attributed to the formation of the Schiff base with the enzyme, as suggested by previous studies on AdSS from *E. coli*^18^. The stabilisation of the AdSS-PLP complex by vitamin C, most possibly through reduction of the Schiff base, is also observed in our experiments. In this regard, the rate constants for the tight-binding complex formation in the presence and absence of 1 mM vitamin C are 14.47 ± 1.89 min^−1^ and 0.037 ± 0.002 min^−1^, respectively.

In addition to the above, our results indicate cooperative binding of PLP with two enzyme substrates, IMP and Asp, as evidenced by the value of the rate constant for this process of 0.73 ± 0.09 min^−1^ in the presence of these substrates. We were also able to prove that PLP competes with the third substrate, GTP, for the same binding site of AdSS from *H. pylori* with the inhibition constant K_i_ = 6.95 ± 0.82 µM. The crystal structure analysis of the AdSS from *H. pylori* in a complex with IMP and PLP, determined and described in this study, confirms the results obtained in solution. The structural data attests that PLP occupies the active site region typically bound by GTP. Moreover, the formation of a covalent bond between PLP and the enzyme, specifically with the side chain of Lys322, is observed, consistent with the formation of the Schiff base (Figures 5, 6 and 2S).

We have also demonstrated that PLP and one of its metabolic precursors, pyridoxal, cause complete inhibition of the *H. pylori* growth in bacterial cultures, and lead to bacterial cells death. This antibacterial effect was observed not only when testing three wild-type *H. pylori* strains (26695, N6 and P12), but also was shown for several clinical strains resistant to metronidazole, clarithromycin or both antibiotics. Interestingly, the resistant strains generally tend to have lower MIC and MBC values as compared with the wild-type strains. Noteworthy, the observed MIC and MBC values for PLP are in the range of 1–2 mM (Table 4), and are significantly higher than IC_50_ = 9.97 µM and IC_50_^eq^ = 0.028 µM, characterising reversible and tight-binding complexes of PLP with AdSS, respectively. The permeability experiments conducted in this study suggest that low uptake of the inhibitor is not responsible for this phenomenon. The possible explanation of this might be that the interaction of PLP with AdSS in the interior of *H. pylori* cells is limited by some process, e.g. by the level of the competing substrate – GTP. Such scenario is possible taking into account a dissociation constant of the GTP-AdSS complex, *K*_d_ = 2.0 ± 0.8 μM^14^. On the other hand, with the present state of knowledge, we are not able to clearly rule out another scenario, in which bacteriostatic and/or bactericidal effects of PLP against *H. pylori* are associated with interaction with other target than only AdSS. Therefore, the question whether inhibition of *H. pylori* AdSS results in stopping the proliferation and eradication of this bacterium remains open. To answer it, a potent *H. pylori* AdSS inhibitor is necessary. Such compound should not occur naturally in this microorganism and should easily enter the bacterial cell. We are currently working on the design and synthesis of such compound and we already have the first promising results (data not shown).

In addition to the above, our results indicate also that checking whether a target for pyridoxal in its original, unphosphorylated form exists in *H. pylori* is worth research verification. The very low penetration of this compound into bacterial cells and the lack of interaction with AdSS suggest that the observed antibacterial effect may result from strong interactions with other cellular targets than AdSS, and have a different, yet unknown mechanism. This is one of the research paths that requires verification in the future.

Parallel to the above considerations, we plan to check if results of this study may be useful in therapies combating *H. pylori*. Although PLP and PI-h can stop the proliferation and thus eradicate this pathogen, the MIC and MBC values observed are high. Even though vitamin B6 is well tolerable by human organism^45^^,^^46^, MIC and MBC of order of 1–2 mM in *in vitro* conditions suggest that doses necessary for *H. pylori* eradication *in vivo* would be too high. However, since all known therapies to combat this pathogen consist of at least two drugs^7^^,^^8^, in the future it would be desirable to determine if such additive or synergistic effect of these medications occurs also together with PLP or PI-h^47^. This will allow for the verification of a proof of concept for replacement of one classically used drug by PLP or PI-h. Alternatively, as PLP is effectively absorbed by *H. pylori* cells from the local environment, this compound may probably serve as a natural vector for the *in vivo* delivery to the cells of these bacteria of biologically active compounds. In line with this idea, vitamin B12 is used for transporting antisense peptide nucleic acid oligomers (PNA) into *E. coli* cells^48^. This type of drug designing uses a “Trojan horse” mechanism, in which one type of molecules vital for the microbial physiology is combined with therapeutical agents^49^. One of the best known examples is cefiderocol, a novel siderophore cephalosporin designed against multidrug-resistant Gram-negative rods^50^.

## Conclusions

In the current study, we have shown that PLP (the active form of vitamin B6) presents antibacterial effect against both reference and clinical strains of *H. pylori*, including those resistant to metronidazole, clarithromycin, and both these antibiotics. This antimicrobial activity is attributed to the formation of a Schiff base with the Lys322 residue of adenylosuccinate synthetase (AdSS) of *H. pylori* and competition with GTP, one of the enzyme substrates. Therefore, antibacterial activity of PLP and its precursor, pyridoxal, together with identification of the AdSS as a target strongly interacting with PLP, suggest several possible practical applications of this compound in designing novel approaches to combat *H. pylori*.

## Supplementary Material

Supplemental Material

## Data Availability

X-ray structure of the PLP in a complex with AdSS and IMP is available in the Protein Data Bank under the code 8QWA. Supplementary data associated with this article can be found in the online version. All other data will be made available upon reasonable request.
