# Supplementary material for: Vitamin B6 inhibits activity of Helicobacter pylori adenylosuccinate synthetase and growth of reference and clinical, antibiotic-resistant H. pylori strains
Source: J Enzyme Inhib Med Chem. 2024 Aug 16;39(1):2372734. doi: 10.1080/14756366.2024.2372734 (PMC11571797; doi:10.1080/14756366.2024.2372734)
Supplement: Supplemental Material [file IENZ_A_2372734_SM1004.pdf]

Supplemental material

Vitamin B6 inhibits activity of *Helicobacter pylori* adenylosuccinate synthetase and growth of reference and clinical, antibiotic-resistant *H. pylori* strains

Marta Ilona Wojtyś[a,b], Weronika Maksymiuk[a], Marta Narczyk[a], Ante Bubić[c], Ivana Leščić Ašler[c], Paweł Krzyżek[d], Grażyna Gościniak[d], Elżbieta Katarzyna Jagusztyn-Krynicka[b], Agnieszka Bzowska[a,*]

[a]*Division of Biophysics, Institute of Experimental Physics, Faculty of Physics, University of Warsaw, Pasteura 5, 02-093 Warsaw, Poland*

[b]*Department of Bacterial Genetics, Institute of Microbiology, Faculty of Biology, University of Warsaw, Miecznikowa 1, 02-096 Warsaw, Poland*

[c]*Division of Physical Chemistry, Ruđer Bošković Institute, Bijenička cesta 54, HR-10000 Zagreb, Croatia*

[d]*Department of Microbiology, Faculty of Medicine, Wroclaw Medical University, Wroclaw 50-368, Poland*

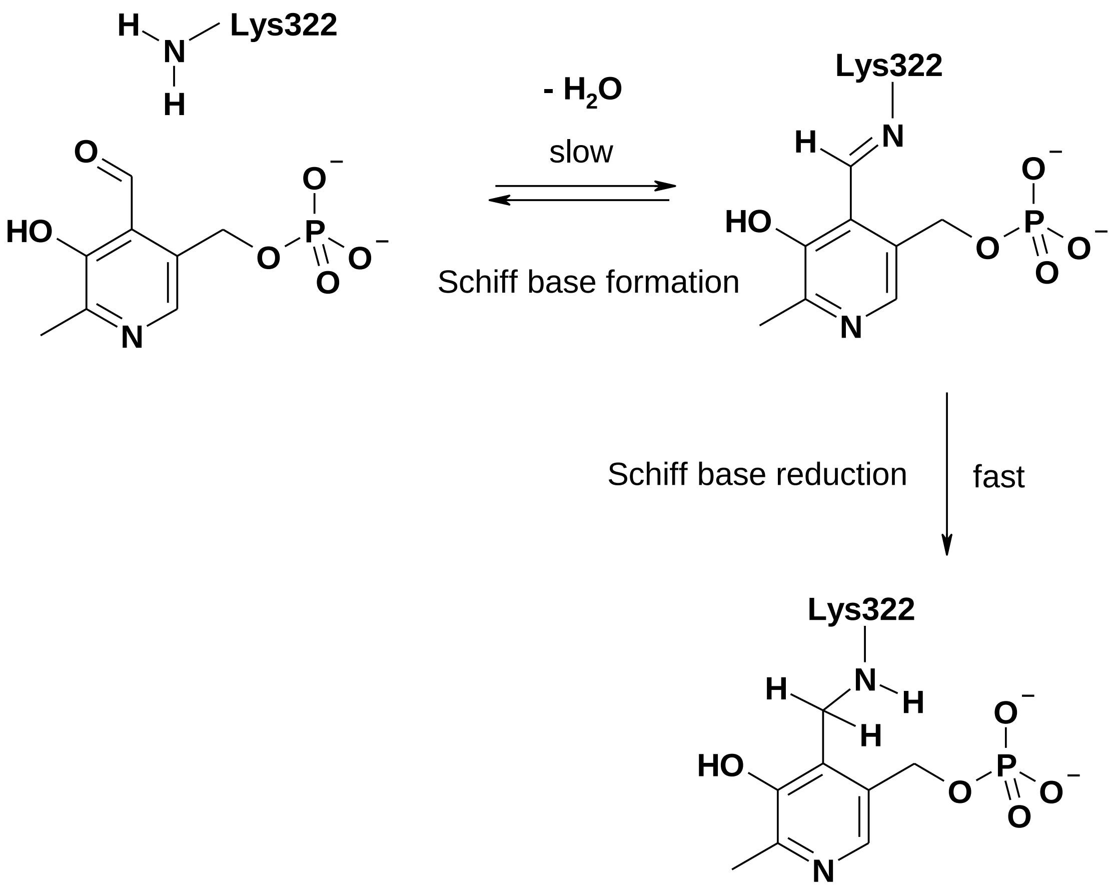

Figure 1S.

Scheme showing the simplified mechanism of the Schiff base formation between pyridoxal 5'-phosphate (PLP) and the side chain of lysine, in the case of *H. pylori* AdSS it is Lys322. Schiff base formation is a slow and reversible process. Stabilization of the complex requires the reduction of the C-N double bond, which may be achieved by a reducing agent such as e.g. vitamin C, which was used in this study.

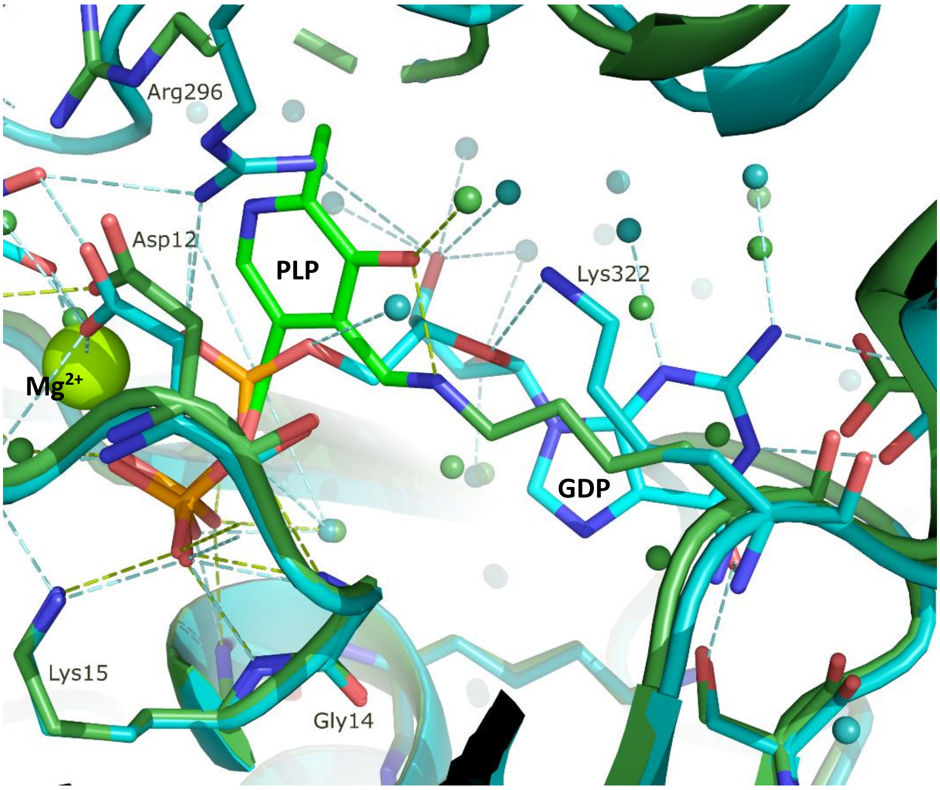

Figure 2S

Active site of the AdSS ternary complex with PLP and IMP (green, 8QWA) overlaid with the active site of the AdSS complex with 6-phosphoryl-IMP, hadacidin, GDP and Mg^{2+} ion (cyan, 6ZXQ)[14]. Zoom on the part where PLP and GDP are bound, respectively in 8QWA and 6ZXQ structures. Mg^{2+} ion is shown as a light green sphere. Hydrogen bond network observed in both complexes are shown with dashed green and cyan lines, respectively. Water molecules are represented by spheres (green and cyan, respectively).

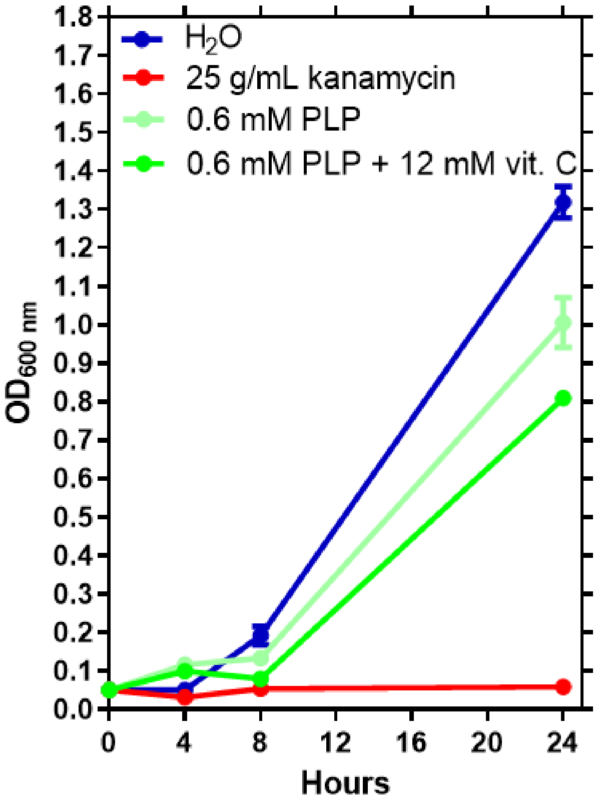

Figure 3S

Growth curves of *H. pylori* 26695 strain in the presence of PLP and vitamin C.
